# (*E*)-6-(4-Chloro­phen­yl)-4-[(2-cyano-3-phenyl­all­yl)sulfan­yl]-2,2-difluoro-3-phenyl-1,3,2-oxaza­borinin-3-ium-2-uide

**DOI:** 10.1107/S1600536813007800

**Published:** 2013-04-05

**Authors:** Ming Li, Shu-Wen Wang, Li-Rong Wen

**Affiliations:** a College of Chemistry and Molecular Engineering, Qingdao University of Science and Technology, Qingdao 266042, People’s Republic of China

## Abstract

In the title compound, C_25_H_18_BClF_2_N_2_OS, the characteristic B—N and B—O bond lengths are 1.571 (3) and 1.458 (3) Å, respectively. The phenyl rings form dihedral angles of 83.1 (1) and 64.6 (1)° with the chloro­phenyl ring. In the crystal, weak C—H⋯N, C—H⋯F, C—H⋯π and π–π inter­actions [centroid–centroid distances 3.877 (6) Å between the chloro­phenyl rings of neighbouring mol­ecules] held mol­ecules together, forming ladders along the *b* axis.

## Related literature

For background to thio­acetanilides, see: Peruncheralathan *et al.* (2005 ▶); Li *et al.* (2010 ▶); Wu *et al.* (2009 ▶); Erten-Ela *et al.* (2008 ▶); Tokoro *et al.* (2010 ▶); Lu *et al.* (2002 ▶); Tsuboyama *et al.* (2003 ▶); Zhang *et al.* (2006 ▶). For the crystal structures of related compounds, see: Macedo *et al.* (2008 ▶).
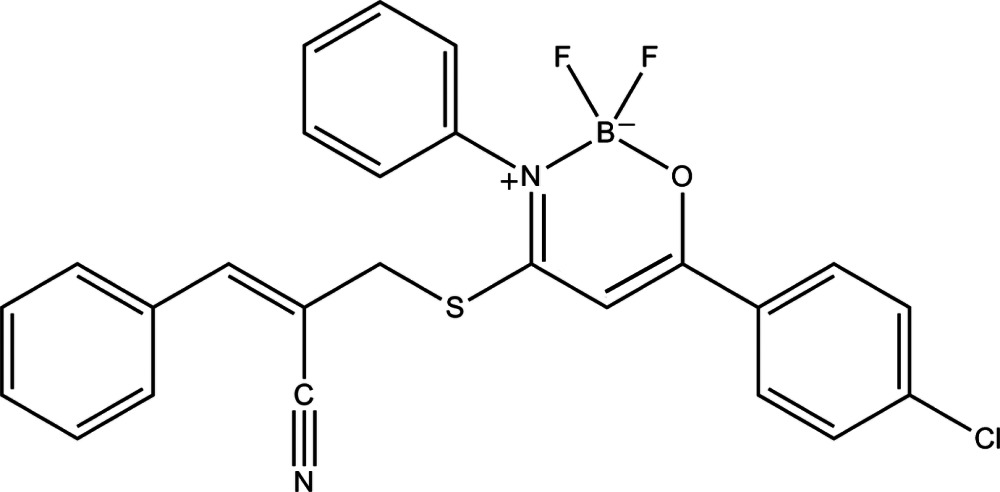


## Experimental

### 

#### Crystal data

C_25_H_18_BClF_2_N_2_OS
*M* *_r_* = 478.73
Monoclinic, 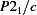

*a* = 9.6996 (19) Å
*b* = 16.290 (3) Å
*c* = 14.168 (3) Å
β = 98.71 (3)°
*V* = 2212.9 (8) Å^3^
*Z* = 4
Mo *K*α radiation
μ = 0.31 mm^−1^
*T* = 173 K
0.43 × 0.29 × 0.09 mm

#### Data collection

Rigaku MM007HF diffractometer with Saturn724+ CCD
Absorption correction: multi-scan (*CrystalClear-SM Expert*; Rigaku/MSC, 2008 ▶) *T*_min_ = 0.685, *T*_max_ = 1.000
15558 measured reflections
5081 independent reflections
4491 reflections with *I* > 2σ(*I*)
*R*_int_ = 0.052

#### Refinement

*R*[*F* ^2^ > 2σ(*F* ^2^)] = 0.058
*wR*(*F* ^2^) = 0.120
*S* = 1.18
5081 reflections
298 parameters
H-atom parameters constrained
Δρ_max_ = 0.31 e Å^−3^
Δρ_min_ = −0.23 e Å^−3^

### 

Data collection: *CrystalClear-SM Expert* (Rigaku/MSC, 2008 ▶); cell refinement: *CrystalClear-SM Expert*; data reduction: *CrystalClear-SM Expert*; program(s) used to solve structure: *SHELXS97* (Sheldrick, 2008 ▶); program(s) used to refine structure: *SHELXL97* (Sheldrick, 2008 ▶); molecular graphics: *SHELXTL* (Sheldrick, 2008 ▶); software used to prepare material for publication: *SHELXTL*.

## Supplementary Material

Crystal structure: contains datablock(s) I, global. DOI: 10.1107/S1600536813007800/cv5394sup1.cif

Structure factors: contains datablock(s) I. DOI: 10.1107/S1600536813007800/cv5394Isup2.hkl

Additional supplementary materials:  crystallographic information; 3D view; checkCIF report

## Figures and Tables

**Table 1:** Hydrogen-bond geometry (Å, °) *Cg*1 is the centroid of the C1–C6 ring.

*D*—H⋯*A*	*D*—H	H⋯*A*	*D*⋯*A*	*D*—H⋯*A*
C16—H16*A*⋯F1^i^	0.99	2.35	3.184 (3)	141
C21—H21⋯N1^ii^	0.95	2.55	3.434 (4)	156
C15—H15⋯*Cg*1^iii^	0.95	2.52	3.394 (5)	153

Symmetry codes: (i) 
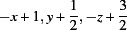
; (ii) 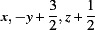; (iii) 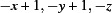.

**Fig. 1.**
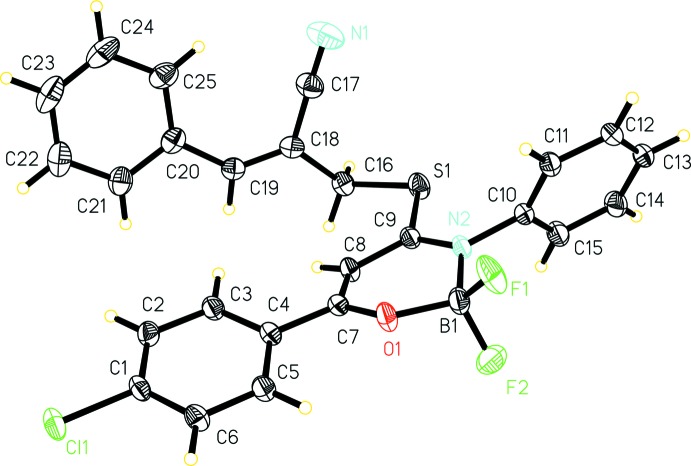
View of (I) showing the atomic numbering and 40% probability displacement ellipsoids.

## supplementary crystallographic information

### Comment

Thioacetanilides being novel enaminones (Peruncheralathan *et al.*, 2005) with four active reaction sites show structural feature of highly polarized push-pull interaction C=C double bond (Li *et al.*, 2010). BF_2_ complexes, such as the Bodipy family, are most intriguing due to their outstanding optical properties, extraordinary chemical versatility, and variety of applications spanning from biolabeling (Wu *et al.*, 2009) to solar cells (Erten-Ela *et al.*, 2008) and nanoparticle engineering (Tokoro *et al.*, 2010). Synthetic studies have been conducted on various phosphorescent materials with heavy metals such as iridium (Tsuboyama *et al.*, 2003) and platinum (Lu *et al.*, 2002) and on fluorescent boron complexes (Zhang *et al.*, 2006). In order to explore the chemical reactivities of thioacetanilides, we obtained the title compound, (I).

In (I) (Fig. 1), the B1–N2 bond length (1.571 (3) Å) is more than 0.1 Å longer than the B1–O1 bond length (1.458 (3) Å) suggesting that the major resonance contribution to bonding is the alkoxy-imine form. All bond lengths and angles in (I) are normal and in a good agreement with those reported previously for related compounds (Macedo *et al.*, 2008) The phenyl rings C10—C15 and C20—C25 form the dihedral angles of 83.1 (1) and 64.6 (1)°, respectively, with the chlorophenyl ring C1—C6.

In the crystal, π-π interactions (centroid-to-centroid distances 3.877 (6) Å between the chlorophenyl rings of neighbouring molecules) and weak intermolecular C—H···N, C—H···F and C—H···π interactions (Table 1) held molecules together.

### Experimental

A 25 ml volumetric flask was charged with 8 ml THF and 1.01 g (5 mmol) 2-cyano-1-phenylallyl acetate. Then 1.45 g (5 mmol) 3-(4-chlorophenyl)-3-oxo-*N*-phenylpropane thioamide and 0.09 g (3.75 mmol) sodium hydride were added. The mixture was stirred at room temperature for 4 h, and then 8 ml BF_3_.Et_2_O (10 mmol) was added with stirring for a further 4 h at room temperature. The reaction was monitored by TLC, and after TLC indicated the completion of the reaction, the solution was filtered, and the inorganic salts were removed. The solvent of the filtrate was removed with the aid of a rotary evaporator, and the residue was purified by column chromatography on silica gel, using petroleum ether/ethyl acetate (6:1) as eluent to provide the desired product yielding 2.01 g (84%) of pure BF2 complex, m.p. 462–463 K. Analytical data: IR (KBr, cm^-1^): 3080, 2977, 2180, 1681, 1583, 1505, 850, 732, 699. ^1^H-NMR (500 MHz, CDCl_3_): δ (p.p.m.) 3.98 (2*H*, s), 6.26 (1*H*, s), 7.22 (1*H*, s), 7.32–7.33 (2*H*, d, J = 7.55 Hz), 7.40–7.49 (8*H*, m), 7.73–7.74 (2*H*, d, J = 7.65 Hz), 7.88–7.90 (2*H*, d, J = 4.28 Hz). Single crystals suitable for X-ray diffraction were obtained through slow evaporation of a solution of the pure title compound in ethanol.

### Refinement

All H atoms were found on difference maps, but placed in idealized positions (C–H = 0.95–0.99 Å), and included in the final cycles of refinement using a riding model, with *U*_iso_(H) = 1.2*U*_eq_(C).

### Crystal data

**Table:**

C_25_H_18_BClF_2_N_2_OS	*F*(000) = 984
*M**_r_* = 478.73	*D*_x_ = 1.437 Mg m^−^^3^
Monoclinic, *P*2_1_/*c*	Mo *K*α radiation, λ = 0.71073 Å
Hall symbol: -P 2ybc	Cell parameters from 7694 reflections
*a* = 9.6996 (19) Å	θ = 1.5–27.5°
*b* = 16.290 (3) Å	µ = 0.31 mm^−^^1^
*c* = 14.168 (3) Å	*T* = 173 K
β = 98.71 (3)°	Plate, colourless
*V* = 2212.9 (8) Å^3^	0.43 × 0.29 × 0.09 mm
*Z* = 4	

### Data collection

**Table:**

Rigaku MM007HF diffractometer with Saturn724+ CCD	5081 independent reflections
Radiation source: Rotating Anode	4491 reflections with *I* > 2σ(*I*)
Confocal monochromator	*R*_int_ = 0.052
Detector resolution: 28.5714 pixels mm^-1^	θ_max_ = 27.5°, θ_min_ = 2.1°
ω scans at fixed χ = 45°	*h* = −12→12
Absorption correction: multi-scan (*CrystalClear-SM Expert*; Rigaku/MSC, 2008)	*k* = −20→21
*T*_min_ = 0.685, *T*_max_ = 1.000	*l* = −18→18
15558 measured reflections	

### Refinement

**Table:**

Refinement on *F*^2^	Primary atom site location: structure-invariant direct methods
Least-squares matrix: full	Secondary atom site location: difference Fourier map
*R*[*F*^2^ > 2σ(*F*^2^)] = 0.058	Hydrogen site location: inferred from neighbouring sites
*wR*(*F*^2^) = 0.120	H-atom parameters constrained
*S* = 1.18	*w* = 1/[σ^2^(*F*_o_^2^) + (0.0312*P*)^2^ + 1.2135*P*] where *P* = (*F*_o_^2^ + 2*F*_c_^2^)/3
5081 reflections	(Δ/σ)_max_ = 0.001
298 parameters	Δρ_max_ = 0.31 e Å^−^^3^
0 restraints	Δρ_min_ = −0.23 e Å^−^^3^

### Special details

**Table:**

Geometry. All e.s.d.'s (except the e.s.d. in the dihedral angle between two l.s. planes) are estimated using the full covariance matrix. The cell e.s.d.'s are taken into account individually in the estimation of e.s.d.'s in distances, angles and torsion angles; correlations between e.s.d.'s in cell parameters are only used when they are defined by crystal symmetry. An approximate (isotropic) treatment of cell e.s.d.'s is used for estimating e.s.d.'s involving l.s. planes.
Refinement. Refinement of *F*^2^ against ALL reflections. The weighted *R*-factor *wR* and goodness of fit *S* are based on *F*^2^, conventional *R*-factors *R* are based on *F*, with *F* set to zero for negative *F*^2^. The threshold expression of *F*^2^ > σ(*F*^2^) is used only for calculating *R*-factors(gt) *etc*. and is not relevant to the choice of reflections for refinement. *R*-factors based on *F*^2^ are statistically about twice as large as those based on *F*, and *R*- factors based on ALL data will be even larger.

### Fractional atomic coordinates and isotropic or equivalent isotropic displacement parameters (Å^2^)

**Table:**

	*x*	*y*	*z*	*U*_iso_*/*U*_eq_	
Cl1	1.18430 (6)	0.47712 (4)	1.29061 (4)	0.03843 (16)	
S1	0.47463 (6)	0.63647 (4)	0.79190 (4)	0.03476 (16)	
F1	0.61480 (16)	0.34159 (9)	0.78320 (10)	0.0494 (4)	
F2	0.46844 (15)	0.35602 (9)	0.89236 (12)	0.0531 (4)	
O1	0.69166 (16)	0.40858 (9)	0.92380 (11)	0.0328 (4)	
N1	0.7666 (3)	0.74393 (18)	0.69117 (18)	0.0609 (7)	
N2	0.51593 (17)	0.47557 (11)	0.80301 (12)	0.0262 (4)	
C1	1.0468 (2)	0.47953 (14)	1.19668 (15)	0.0274 (5)	
C2	1.0082 (2)	0.55340 (14)	1.15244 (16)	0.0304 (5)	
H2	1.0558	0.6027	1.1730	0.036*	
C3	0.8982 (2)	0.55382 (13)	1.07731 (16)	0.0292 (5)	
H3	0.8696	0.6041	1.0465	0.035*	
C4	0.8293 (2)	0.48155 (13)	1.04648 (15)	0.0250 (4)	
C5	0.8714 (2)	0.40787 (13)	1.09205 (16)	0.0292 (5)	
H5	0.8256	0.3582	1.0709	0.035*	
C6	0.9795 (2)	0.40674 (14)	1.16804 (16)	0.0299 (5)	
H6	1.0071	0.3568	1.2000	0.036*	
C7	0.7153 (2)	0.48088 (13)	0.96488 (15)	0.0248 (4)	
C8	0.6431 (2)	0.54917 (13)	0.93219 (15)	0.0275 (5)	
H8	0.6567	0.5989	0.9674	0.033*	
C9	0.5476 (2)	0.54684 (13)	0.84604 (15)	0.0261 (4)	
C10	0.4192 (2)	0.47173 (13)	0.71416 (15)	0.0264 (4)	
C11	0.4659 (2)	0.44916 (14)	0.63062 (15)	0.0299 (5)	
H11	0.5618	0.4376	0.6303	0.036*	
C12	0.3708 (2)	0.44347 (14)	0.54681 (16)	0.0332 (5)	
H12	0.4020	0.4278	0.4890	0.040*	
C13	0.2318 (2)	0.46038 (15)	0.54715 (17)	0.0353 (5)	
H13	0.1674	0.4568	0.4897	0.042*	
C14	0.1866 (2)	0.48238 (16)	0.63088 (17)	0.0371 (6)	
H14	0.0907	0.4944	0.6309	0.044*	
C15	0.2795 (2)	0.48729 (15)	0.71564 (17)	0.0351 (5)	
H15	0.2475	0.5012	0.7737	0.042*	
C16	0.5544 (2)	0.71958 (13)	0.86749 (16)	0.0305 (5)	
H16A	0.4994	0.7701	0.8507	0.037*	
H16B	0.5464	0.7061	0.9346	0.037*	
C17	0.7407 (2)	0.74202 (16)	0.76730 (19)	0.0392 (6)	
C18	0.7058 (2)	0.73849 (13)	0.86224 (16)	0.0290 (5)	
C19	0.7974 (2)	0.75408 (13)	0.94089 (17)	0.0298 (5)	
H19	0.7598	0.7497	0.9988	0.036*	
C20	0.9453 (2)	0.77676 (13)	0.95341 (18)	0.0331 (5)	
C21	1.0184 (3)	0.77306 (15)	1.04606 (19)	0.0399 (6)	
H21	0.9705	0.7581	1.0972	0.048*	
C22	1.1595 (3)	0.79076 (17)	1.0647 (2)	0.0497 (7)	
H22	1.2079	0.7870	1.1281	0.060*	
C23	1.2294 (3)	0.81371 (17)	0.9917 (3)	0.0549 (8)	
H23	1.3263	0.8257	1.0045	0.066*	
C24	1.1594 (3)	0.81935 (16)	0.8998 (2)	0.0525 (8)	
H24	1.2083	0.8351	0.8494	0.063*	
C25	1.0182 (3)	0.80214 (15)	0.8805 (2)	0.0412 (6)	
H25	0.9703	0.8076	0.8172	0.049*	
B1	0.5719 (3)	0.39255 (16)	0.85061 (19)	0.0319 (6)	

### Atomic displacement parameters (Å^2^)

**Table:**

	*U*^11^	*U*^22^	*U*^33^	*U*^12^	*U*^13^	*U*^23^
Cl1	0.0282 (3)	0.0561 (4)	0.0277 (3)	0.0032 (2)	−0.0063 (2)	−0.0049 (3)
S1	0.0339 (3)	0.0328 (3)	0.0334 (3)	0.0042 (2)	−0.0084 (2)	0.0026 (2)
F1	0.0668 (10)	0.0414 (8)	0.0339 (8)	0.0178 (7)	−0.0125 (7)	−0.0125 (6)
F2	0.0451 (9)	0.0497 (9)	0.0622 (11)	−0.0141 (7)	0.0003 (7)	0.0157 (8)
O1	0.0389 (9)	0.0252 (8)	0.0294 (9)	0.0015 (7)	−0.0102 (7)	−0.0042 (6)
N1	0.0547 (15)	0.090 (2)	0.0404 (15)	0.0242 (14)	0.0130 (11)	0.0166 (13)
N2	0.0249 (9)	0.0321 (10)	0.0201 (9)	−0.0005 (7)	−0.0013 (7)	−0.0020 (7)
C1	0.0232 (10)	0.0402 (12)	0.0181 (11)	0.0031 (9)	0.0005 (8)	−0.0029 (9)
C2	0.0307 (11)	0.0330 (12)	0.0266 (12)	−0.0051 (9)	0.0020 (9)	−0.0050 (9)
C3	0.0328 (11)	0.0278 (11)	0.0256 (12)	−0.0010 (9)	−0.0003 (9)	−0.0007 (9)
C4	0.0265 (10)	0.0281 (11)	0.0197 (11)	0.0020 (8)	0.0014 (8)	−0.0020 (8)
C5	0.0317 (11)	0.0278 (11)	0.0266 (12)	0.0011 (9)	−0.0004 (8)	−0.0011 (9)
C6	0.0325 (11)	0.0287 (11)	0.0265 (12)	0.0060 (9)	−0.0014 (9)	−0.0004 (9)
C7	0.0277 (10)	0.0263 (11)	0.0202 (11)	−0.0026 (8)	0.0029 (8)	−0.0011 (8)
C8	0.0294 (11)	0.0279 (11)	0.0229 (11)	0.0006 (9)	−0.0029 (8)	−0.0035 (8)
C9	0.0246 (10)	0.0308 (11)	0.0222 (11)	−0.0003 (8)	0.0011 (8)	−0.0006 (8)
C10	0.0266 (10)	0.0297 (11)	0.0214 (11)	−0.0042 (9)	−0.0011 (8)	0.0017 (8)
C11	0.0271 (11)	0.0366 (12)	0.0251 (12)	−0.0038 (9)	0.0014 (8)	−0.0026 (9)
C12	0.0390 (12)	0.0392 (13)	0.0210 (12)	−0.0095 (10)	0.0036 (9)	−0.0018 (9)
C13	0.0352 (12)	0.0430 (14)	0.0245 (12)	−0.0097 (10)	−0.0061 (9)	0.0050 (10)
C14	0.0254 (11)	0.0512 (15)	0.0326 (13)	−0.0026 (10)	−0.0020 (9)	0.0011 (11)
C15	0.0278 (11)	0.0507 (15)	0.0267 (13)	−0.0038 (10)	0.0035 (9)	−0.0049 (10)
C16	0.0297 (11)	0.0288 (11)	0.0317 (13)	0.0074 (9)	0.0002 (9)	0.0004 (9)
C17	0.0345 (12)	0.0473 (15)	0.0353 (15)	0.0106 (11)	0.0041 (10)	0.0065 (11)
C18	0.0318 (11)	0.0246 (10)	0.0311 (12)	0.0071 (9)	0.0066 (9)	0.0030 (9)
C19	0.0331 (11)	0.0248 (11)	0.0317 (13)	0.0024 (9)	0.0058 (9)	0.0010 (9)
C20	0.0330 (11)	0.0214 (10)	0.0449 (15)	0.0013 (9)	0.0061 (10)	0.0002 (10)
C21	0.0380 (13)	0.0353 (13)	0.0452 (16)	−0.0021 (10)	0.0029 (11)	−0.0054 (11)
C22	0.0386 (14)	0.0444 (15)	0.0616 (19)	−0.0029 (12)	−0.0067 (12)	−0.0058 (13)
C23	0.0334 (13)	0.0383 (15)	0.092 (3)	−0.0078 (12)	0.0072 (14)	−0.0011 (15)
C24	0.0411 (14)	0.0341 (14)	0.086 (2)	−0.0042 (12)	0.0215 (15)	0.0109 (14)
C25	0.0397 (13)	0.0314 (13)	0.0536 (17)	0.0016 (10)	0.0105 (11)	0.0096 (11)
B1	0.0342 (13)	0.0305 (13)	0.0284 (14)	−0.0032 (11)	−0.0040 (10)	−0.0041 (10)

### Geometric parameters (Å, º)

**Table:**

Cl1—C1	1.736 (2)	C11—C12	1.392 (3)
S1—C9	1.748 (2)	C11—H11	0.9500
S1—C16	1.824 (2)	C12—C13	1.377 (3)
F1—B1	1.376 (3)	C12—H12	0.9500
F2—B1	1.375 (3)	C13—C14	1.373 (3)
O1—C7	1.318 (2)	C13—H13	0.9500
O1—B1	1.458 (3)	C14—C15	1.391 (3)
N1—C17	1.144 (3)	C14—H14	0.9500
N2—C9	1.326 (3)	C15—H15	0.9500
N2—C10	1.453 (3)	C16—C18	1.513 (3)
N2—B1	1.571 (3)	C16—H16A	0.9900
C1—C2	1.382 (3)	C16—H16B	0.9900
C1—C6	1.384 (3)	C17—C18	1.437 (3)
C2—C3	1.388 (3)	C18—C19	1.340 (3)
C2—H2	0.9500	C19—C20	1.466 (3)
C3—C4	1.391 (3)	C19—H19	0.9500
C3—H3	0.9500	C20—C21	1.396 (3)
C4—C5	1.394 (3)	C20—C25	1.400 (3)
C4—C7	1.474 (3)	C21—C22	1.384 (3)
C5—C6	1.385 (3)	C21—H21	0.9500
C5—H5	0.9500	C22—C23	1.372 (4)
C6—H6	0.9500	C22—H22	0.9500
C7—C8	1.358 (3)	C23—C24	1.377 (4)
C8—C9	1.417 (3)	C23—H23	0.9500
C8—H8	0.9500	C24—C25	1.384 (4)
C10—C11	1.380 (3)	C24—H24	0.9500
C10—C15	1.381 (3)	C25—H25	0.9500
			
C9—S1—C16	104.75 (10)	C13—C14—C15	120.7 (2)
C7—O1—B1	122.73 (17)	C13—C14—H14	119.6
C9—N2—C10	120.69 (18)	C15—C14—H14	119.6
C9—N2—B1	121.04 (17)	C10—C15—C14	119.0 (2)
C10—N2—B1	118.08 (17)	C10—C15—H15	120.5
C2—C1—C6	121.98 (19)	C14—C15—H15	120.5
C2—C1—Cl1	119.43 (17)	C18—C16—S1	116.79 (16)
C6—C1—Cl1	118.60 (17)	C18—C16—H16A	108.1
C1—C2—C3	118.4 (2)	S1—C16—H16A	108.1
C1—C2—H2	120.8	C18—C16—H16B	108.1
C3—C2—H2	120.8	S1—C16—H16B	108.1
C2—C3—C4	120.9 (2)	H16A—C16—H16B	107.3
C2—C3—H3	119.6	N1—C17—C18	178.8 (3)
C4—C3—H3	119.6	C19—C18—C17	123.4 (2)
C3—C4—C5	119.35 (19)	C19—C18—C16	121.6 (2)
C3—C4—C7	121.36 (19)	C17—C18—C16	115.0 (2)
C5—C4—C7	119.27 (19)	C18—C19—C20	131.4 (2)
C6—C5—C4	120.4 (2)	C18—C19—H19	114.3
C6—C5—H5	119.8	C20—C19—H19	114.3
C4—C5—H5	119.8	C21—C20—C25	117.7 (2)
C1—C6—C5	119.0 (2)	C21—C20—C19	116.8 (2)
C1—C6—H6	120.5	C25—C20—C19	125.4 (2)
C5—C6—H6	120.5	C22—C21—C20	121.2 (3)
O1—C7—C8	122.33 (19)	C22—C21—H21	119.4
O1—C7—C4	114.39 (18)	C20—C21—H21	119.4
C8—C7—C4	123.25 (19)	C23—C22—C21	120.0 (3)
C7—C8—C9	120.5 (2)	C23—C22—H22	120.0
C7—C8—H8	119.7	C21—C22—H22	120.0
C9—C8—H8	119.7	C22—C23—C24	120.1 (3)
N2—C9—C8	119.69 (19)	C22—C23—H23	119.9
N2—C9—S1	118.65 (15)	C24—C23—H23	119.9
C8—C9—S1	121.61 (16)	C23—C24—C25	120.3 (3)
C11—C10—C15	120.8 (2)	C23—C24—H24	119.9
C11—C10—N2	120.13 (19)	C25—C24—H24	119.9
C15—C10—N2	119.0 (2)	C24—C25—C20	120.7 (3)
C10—C11—C12	119.2 (2)	C24—C25—H25	119.7
C10—C11—H11	120.4	C20—C25—H25	119.7
C12—C11—H11	120.4	F2—B1—F1	110.8 (2)
C13—C12—C11	120.4 (2)	F2—B1—O1	109.0 (2)
C13—C12—H12	119.8	F1—B1—O1	108.0 (2)
C11—C12—H12	119.8	F2—B1—N2	109.24 (19)
C14—C13—C12	119.8 (2)	F1—B1—N2	110.0 (2)
C14—C13—H13	120.1	O1—B1—N2	109.71 (18)
C12—C13—H13	120.1		
			
C6—C1—C2—C3	0.2 (3)	C10—C11—C12—C13	0.2 (3)
Cl1—C1—C2—C3	179.82 (17)	C11—C12—C13—C14	−0.5 (4)
C1—C2—C3—C4	−0.7 (3)	C12—C13—C14—C15	−0.4 (4)
C2—C3—C4—C5	0.2 (3)	C11—C10—C15—C14	−2.0 (4)
C2—C3—C4—C7	−178.1 (2)	N2—C10—C15—C14	−179.0 (2)
C3—C4—C5—C6	0.8 (3)	C13—C14—C15—C10	1.6 (4)
C7—C4—C5—C6	179.1 (2)	C9—S1—C16—C18	72.85 (18)
C2—C1—C6—C5	0.8 (3)	N1—C17—C18—C19	157 (14)
Cl1—C1—C6—C5	−178.86 (17)	N1—C17—C18—C16	−26 (14)
C4—C5—C6—C1	−1.3 (3)	S1—C16—C18—C19	−137.62 (19)
B1—O1—C7—C8	−10.5 (3)	S1—C16—C18—C17	45.1 (2)
B1—O1—C7—C4	171.5 (2)	C17—C18—C19—C20	−0.2 (4)
C3—C4—C7—O1	158.4 (2)	C16—C18—C19—C20	−177.3 (2)
C5—C4—C7—O1	−19.9 (3)	C18—C19—C20—C21	−167.7 (2)
C3—C4—C7—C8	−19.6 (3)	C18—C19—C20—C25	12.7 (4)
C5—C4—C7—C8	162.2 (2)	C25—C20—C21—C22	−2.5 (4)
O1—C7—C8—C9	−6.2 (3)	C19—C20—C21—C22	177.9 (2)
C4—C7—C8—C9	171.6 (2)	C20—C21—C22—C23	1.1 (4)
C10—N2—C9—C8	−179.45 (19)	C21—C22—C23—C24	0.1 (4)
B1—N2—C9—C8	5.6 (3)	C22—C23—C24—C25	0.2 (4)
C10—N2—C9—S1	−1.9 (3)	C23—C24—C25—C20	−1.7 (4)
B1—N2—C9—S1	−176.82 (16)	C21—C20—C25—C24	2.8 (4)
C7—C8—C9—N2	8.1 (3)	C19—C20—C25—C24	−177.7 (2)
C7—C8—C9—S1	−169.38 (17)	C7—O1—B1—F2	−97.9 (2)
C16—S1—C9—N2	−175.52 (17)	C7—O1—B1—F1	141.6 (2)
C16—S1—C9—C8	2.0 (2)	C7—O1—B1—N2	21.6 (3)
C9—N2—C10—C11	112.8 (2)	C9—N2—B1—F2	100.3 (2)
B1—N2—C10—C11	−72.1 (3)	C10—N2—B1—F2	−74.8 (3)
C9—N2—C10—C15	−70.1 (3)	C9—N2—B1—F1	−137.9 (2)
B1—N2—C10—C15	105.0 (2)	C10—N2—B1—F1	47.0 (3)
C15—C10—C11—C12	1.1 (3)	C9—N2—B1—O1	−19.2 (3)
N2—C10—C11—C12	178.1 (2)	C10—N2—B1—O1	165.75 (18)

### Hydrogen-bond geometry (Å, º)

Cg1 is the centroid of the C1–C6 ring.

**Table:**

*D*—H···*A*	*D*—H	H···*A*	*D*···*A*	*D*—H···*A*
C16—H16*A*···F1^i^	0.99	2.35	3.184 (3)	141
C21—H21···N1^ii^	0.95	2.55	3.434 (4)	156
C15—H15···*Cg*1^iii^	0.95	2.52	3.394 (5)	153

Symmetry codes: (i) −*x*+1, *y*+1/2, −*z*+3/2; (ii) *x*, −*y*+3/2, *z*+1/2; (iii) −*x*+1, −*y*+1, −*z*.
